# Effects of Biochar Addition on the Properties of Alkali-Activated Materials

**DOI:** 10.3390/ma18030486

**Published:** 2025-01-21

**Authors:** Andrea Saccani, Luca Baldazzi, Stefania Manzi

**Affiliations:** Department of Civil, Chemical, Environmental and Materials Engineering, University of Bologna, Via Terracini 28, 40131 Bologna, Italy; luca.baldazzi4@unibo.it (L.B.); stefania.manzi4@unibo.it (S.M.)

**Keywords:** alkali-activated materials, biochar, mechanical properties, durability, metakaolin

## Abstract

The addition of biochar to Portland cement composites has been proven to increase some of the material properties. The effect on alkali-activated materials has not been fully investigated. In this study, different recipes of metakaolin pastes at different biochar amounts are tested. Their physical and mechanical properties are analyzed to understand if any beneficial effects can be found even for alkali-activated binders. The results show that the addition of small amounts of biochar (<2 wt%) increases the compressive strength of metakaolin pastes (+15% after 28 days) and decreases the water absorption by capillarity, possibly leading to increased durability. Higher biochar content decreases the mechanical properties but provides higher dimensional stability and reduces the formation of efflorescence.

## 1. Introduction

In 2005 the atmospheric CO_2_ level was 280 ppm, and in 2019, it was 409.8 ppm. This increase was caused mainly by manufacturing industries, and among them, construction-based industries had an important impact on global CO_2_ emissions (around 7%) [1]. To achieve the goal of reducing the emission of greenhouse gases into the atmosphere, in recent years, many studies have been conducted to reduce the amount of clinker added to concrete, partially replacing it with other materials called supplementary cementitious materials (SCMs) coming from industrial wastes or construction and demolition wastes (CDWs). A completely alternative approach makes use of different binders. These materials are obtained starting from a solid alumino-silicate precursor that reacts with different strongly alkaline activators to create a 3D structure with high mechanical strength. The chemical reactions involved are consequently entirely different from those active in Portland cement materials. The precursors must be rich in silica and alumina, such as fly ash, silica fume, metakaolin, or blast furnace slag [2]. Independently from the source, alkali-activated materials generate lower CO_2_ emissions than traditional Portland cement. These materials can be defined either as alkali-activated materials (AAMs) or as geopolymers on account of their overall chemical composition [3]. On the other hand, biochar is obtained from an organic substance (generally agricultural wastes). The average CO_2_ footprint is up to −3.3 kg of CO_2_-eq per kg of biochar [4], confirming its capability to absorb atmospheric CO_2_ (reduction in greenhouse gases near 900 kg CO_2_) [5]. Accordingly, the addition of biochar into building materials can minimize their carbon footprint since biochar is a carbon-negative material. Gupta tested peanut hull biochar [6] and wood sawdust biochar [7], finding a value of 0.86 mmol/g of CO_2_ adsorption capacity for peanut hull and 1.67 mmol/g for wood sawdust. Furthermore, replacing 2 wt% of cement with wood sawdust biochar in mortars reduces the net global warming potential by 15.3%. Four different processes can be applied to obtain biochar: slow and fast pyrolysis, microwave pyrolysis, and gasification. In the first one, the organic matter is heated at 500 °C with a speed rate of 10 °C·min^−1^; in the fast pyrolysis, fine biomass (<1 mm) is heated at 400–800 °C at high speed for a short time, resulting in incomplete lignin depolymerization [5]. Microwave pyrolysis uses microwave energy to heat the biomass, resulting in a more precise temperature control programme and the production of high-quality biochar showing higher surface area and porosity, which is very useful for removing contaminants from polluted water [8]. Gasification can be performed using steam or hot and compressed water and the products are chars and gases such as hydrogen, carbon mono- and dioxide, and methane [4,5]. Currently, biochar is mainly used as a soil conditioner to improve the performance of the soil [8]. Its final properties are related to different factors such as the nature of biomass, moisture content, manufacturing process, and temperature of pyrolysis. The elements generally present in biochar are C, H, O, N, and a few mineral elements (K, Ca, Mg, and Na) [4]. The original biomass also affects the biochar pore structure: woody biochar has a higher pore structure compared to other types of biochar, increasing water retention capacity [4]. Biochar also has high heat resistance, due to its covalent structure and aromaticity, low thermal conductivity, and high resistance to microbial degradation [4].

In recent years, articles and reviews have been published about the use of biochar as a partial replacement for Portland cement [9,10]. For example, Maljaee in his review [11] found that the optimum biochar addition is 2 wt% to improve mechanical properties. Porous biochar absorbs, in the first stage of the sample preparation, a part of the mixing water, reducing the effective water-to-binder ratio. During this time, the absorbed water is released, maintaining a constant internal moisture content and exerting a curing action [5,6,7,8,9,10,11]. Barbhuiya also came to similar conclusions about the improvement of mechanical properties, finding the best addition near 1–5 wt% of biochar [8]. Biochar is also able to improve the mechanical interlocking with the cement matrix. The amount of biochar to add depends on the feedstock type and particle size [1]. Chen [4] compared research where biochar was added to cement binder finding the best mechanical performance in the use of sludge and rice hush biochar at 5 wt% replacement; however, in [12], the incorporation of 2 wt% sewage sludge biochar pyrolyzed at 500 °C to Portland cement gave an increase in compressive strength of 5.8% after 28 days (near 60 MPa).

Gupta [13] tested the substitution with wood biochar (1–2 wt%) and rice husk biochar (2 wt%), obtaining an increase in mortar strength by 17–24%. These results are also confirmed in [14] where the addition of 0.5–1 wt% of mixed wood sawdust biochar (0.1–2 μm) increased the compressive strength by 20%.

Gupta [15] also tested the possibility of replacing cement with biochar made with wood sawdust, finding that 1–2 wt% is the optimum amount. The main reason for this improvement is supposed to be the bonding capability with the cementitious matrix, thanks to the presence of a ridge-shaped surface and a porous structure in the biochar. Furthermore, these amounts do not significantly reduce flowability and density compared to the control mix mortar. Replacement near 5–8 wt% in reverse reduces the flowability and creates more voids that increase porosity and reduce compressive strength too [15]. Biochar particles, absorbing part of free water, reduce the local water–cement ratio and create a denser mortar. Wang [16], Gupta [13], and Muthukrishnan [17] found that the addition of biochar particles creates a “reservoir effect” that leads to a denser microstructure during the secondary hydration process. The main phases of this process are attraction by the biochar of cement grains through hydroxyl functional groups, formation of clusters, nucleation, attraction of other biochar, precipitation of hydration products on the surfaces of the clusters, and densification of the cement matrix. De Carvalho [18] obtained pastes by mixing cement and biochar coming from water treatment sludge (1–2–5 wt%), observing that the concentration of C–S–H and portlandite is very similar to the reference material, in terms of the compressive strength. These results are reached thanks to the partial pozzolanic effect of biochar due to the presence of metakaolinite. The densification was also confirmed by SEM analysis where the microstructure of the pastes with additions of 1 wt% and 2 wt% biochar showed a well-formed structure with a network of C–S–H compounds [18]. Chen [12] studied the hydration mechanisms of sludge biochar and cement composed of three simultaneous phases: nucleation and crystal growth, interactions at phase boundaries, and diffusion. Zeidabadi [19], testing the addition of rice husk and sugarcane bagasse pyrolyzed at 700 °C, found that additions above 5 wt% lead to a reduction in mechanical properties. Navaratnam [20] tested the addition of wood biochar pyrolyzed at 500 °C to prepare cement mortars exposed to high temperatures (from 200 °C to 700 °C), finding that the best performance was obtained with a 5 wt% addition; for higher dosage levels (10–20 wt%), a decrease around 19–53% in compressive strength was obtained.

The abundance of papers dealing with biochar in addition to traditional Portland materials stresses the large interest in this practice. Few studies in the literature are found on the addition of biochar to alkali-activated materials (AAMs) or geopolymers [21,22,23,24,25,26,27,28,29,30,31,32,33,34,35,36,37,38,39,40]. Recently, various applications in different sectors have been proposed for the removal of heavy metals, particularly lead [21,22,23,24,25,26,27,28,29,30,31,32,33], and polluting substances such as methylene blue [24], rhodamine B [21], naphthalene [25], and also carbon dioxide absorbers [26,27]. In particular, Chen [4] foresees an increase in the use of solid waste applied to the construction sector in addition to red mud, phosphogypsum, and alkali-activated binders. The hydration products, either ettringite or C-A-S-H, and biochar can encapsulate the pollutants, restraining them thanks to ion exchange, redox reactions, pore filling, electrostatic attraction, and physical encapsulation [4]. Its good insulating properties make it a suitable material for the production of insulating panels [28] or foams [29], and its low weight makes it suitable for creating lightweight materials for building construction [28].

As for the production of mortars and concrete, Egodagamage [30] performed a mix design of granulated blast furnace slag AAM, investigating the effect of biochar type, amount, and alkaline activator type and determining the formulation for achieving the best mechanical results. Biochar deriving from rice husk was tested by Zhao [31] in addition to fly ash and slag activated with sodium hydroxide and a sodium silicate solution. Rice husk has many interconnected pores so its introduction creates a product with lighter mass, higher water absorption, and a better capillary absorption coefficient. The pores in the matrix reduce the progression of water evaporation and the presence of efflorescence. Moreover, the reactive SiO_2_ present in rice husk promotes the formation of N-A-S-H and C-(A)-S-H gels together with alkali activators [31].

Prabahar [32] tested geopolymeric pastes with blast furnace slag and biochar at 1.54% in a NaOH solution and 1.63% in a Na_2_CO_3_ solution. The pastes with biochar had reduced autogenous shrinkage (in particular, for the ones activated with the NaOH solution). Compressive strength was increased in the pastes containing biochar thanks to the absorption of the activator solution made by biochar that created a denser pore structure. It should also be considered that the presence of biochar voids reduces the compressive strength since they act as stress concentration sites in the sample. Thus, it is supposed that the effect of densification compensated for the effect of voids since the pastes with biochar showed an increase in compressive strength after 7 and 24 days [32]. Table 1 summarizes the previous papers found in the literature concerning the use of biochar in addition to traditional Portland materials [4,8,11,12,13,14,15,16,17,18,19,20] and AAMs [21,28,29,30,31,32,33,34,35,36,37,38,39,40], as well as the characterization that was made on the composite materials (such as paste (P), mortar (M), and concrete (C)), with a particular focus on the mechanical (compressive strength (σ_c_)) and durability properties.

So far, the role and the effects of biochar on this class of materials, AAMs, which have peculiar properties compared to traditional Portland composites, are far from being completely examined and understood. In the present research, the effect of different biochar amounts on the overall performance of pastes containing metakaolin and activated by sodium hydroxide and sodium silicate has been investigated.

## 2. Materials and Methods

### 2.1. Materials and Recipes

The metakaolin used was obtained by rapid calcination of a kaolinitic clay with an original production process that requires low energy consumption and low CO_2_ emission. Its commercial name is Argicem and it is produced by Argeco Développement located in Fumel (Lot-et-Garonne), France. The density of metakaolin measured using a helium pycnometer (Ultrapycnometer 1000, Quantachrome Instruments, Boynton Beach, FL, USA) was equal to 2.627 g/cm^3^ and its specific surface area is 10.48 m^2^/g (BET analysis, Nova 800, Anton Paar, Graz, Austria). The X-ray analysis of metakaolin has been previously reported [41]. The diffractogram showed the presence of silica. To activate the metakaolin, an 8M solution of NaOH (Merck, Darmstadt, Germany) was used in combination with a Na_2_SiO_3_ solution (commercial name Reoflux B, SS, Ingessil, Verona, Italy) with the following characteristics: water content = 56 wt%; SiO_2_/Na_2_O ratio = 2.07; and ρ = 1.53 g/cm^3^.

**Figure 1:**
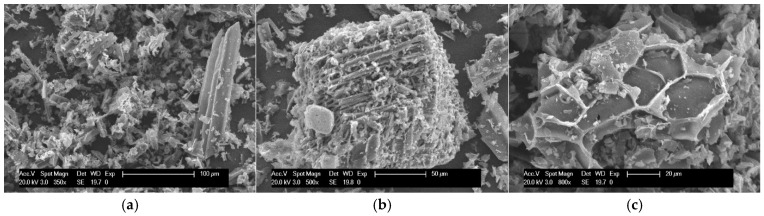
(**a**–**c**) Morphology of biochar.

The biochar used to prepare the pastes comes from non-contaminated chipped hardwood (mainly peach and grapevine) slowly pyrolyzed (from ambient temperature to 550 °C with a heating rate of 10–15 °C·min^−1^ followed by an isothermal condition of 550 °C for 30 min). This biochar was produced by Romagna Carbone snc, located in Bagnacavallo (RA), Italy, and kindly provided by the Department of Agricultural Sciences of the University of Bologna, Italy [33]. The elemental chemical composition of biochar is reported in [33]. Biochar was previously dried in the oven at 55 °C for 72 h, and later, it was manually ground and sieved using a sieve of ϕ 0.5 mm. The density of the biochar measured using the helium pycnometer was 1.961 g/cm^3^, with a specific surface area of 5.73 m^2^/g and a volume porosity of 5%. Figure 1a–c show the morphology of the filler obtained by scanning electron microscopy (FEI Instruments, FEI, Hillsboro, OR, USA). Biochar particles show a very complex shape, rough surface, and a large size distribution. The larger particles (Figure 1b) disclose a porous network capable of absorbing water, while some of them show organized hexagonal microfibrils that belong to the lignocellulosic materials (grapevine).

**Figure 2:**
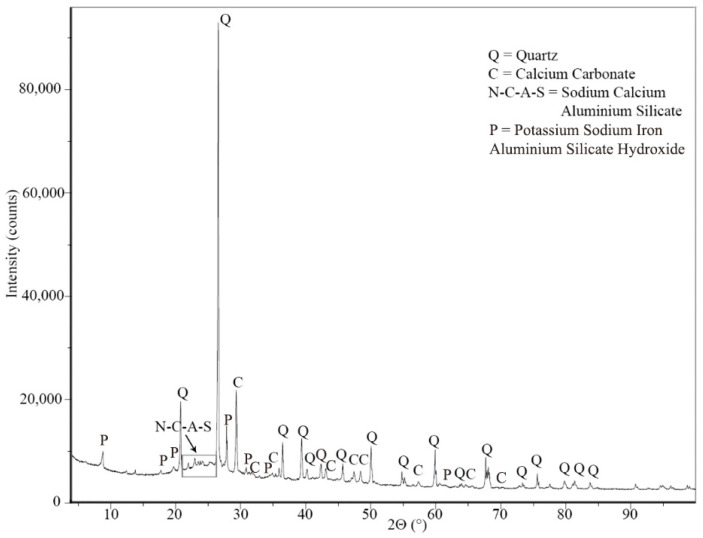
XRD of the biochar.

Moreover, biochar was analyzed with the XRD technique (diffractometer Empyrean, Malvern Panalytical, detector PIXcel1D, Almelo, The Netherlands) to determine its composition. The main elements detected are quartz, calcium carbonate, and sodium calcium aluminum silicate (Figure 2). A small peak is also present at a low 2θ value that can probably be assigned to potassium sodium iron aluminum silicate hydroxide.

Comparing the XRD results with others present in the literature, it is possible to observe the presence of amorphous and crystalline phases of silica. For example, Muthukrishnan [17], Gupta [13], and Egodagamage [30] found, for rice husk biochar, sharp and narrow peaks at 2θ = 22°, indicating the presence of crystalline phases of silica, and a hump between 20 and 25° belonging to amorphous silica. This one can have a pozzolanic effect during the hydration process, even with a limited contribution to the final geopolymerization.

**Figure 3:**
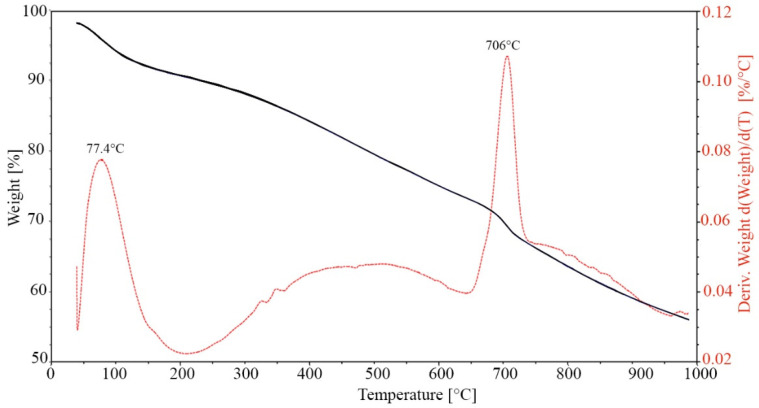
TGA graphic of the biochar used.

The biochar was also analyzed by thermogravimetric analysis (TGA 55, TA Instruments, Waters LLC, New Castel, DE 19720, USA) with the following method: ramp 20 °C·min^−1^ to 1000 °C (Figure 3). The graphic obtained discloses a steady and constant decomposition leading to an almost linear decrease in weight loss. However, a small peak appears at 706 °C that may be related to the decomposition of calcium carbonate (corresponding to about 20% of the total mass of biochar).

### 2.2. Mix Design and Sample Preparation

The formulated recipes are reported in Table 2. Starting from an unmodified metakaolin composition (MH-0), different mix designs were investigated with increasing amounts of biochar substituting 1, 2, 5, and 8 wt% of metakaolin (MH-1, MH-2, MH-5, and MH-8). The quantity of activators was kept constant although this caused a slight unbalance in the ratio among the different oxides of silicium, aluminum, and sodium. Alkali-activated pastes with biochar were obtained by using a beaker (1 L volume) and a mixer (Janke & Kunkel IKA: Labortechnik RW-20, Haverhill, MA, USA). After weighing the ingredients, metakaolin and biochar were placed in the beaker, and later, the activation solution (NaOH and Na_2_SiO_3_, previously mixed) was poured in. Finally, water was also added to the mix. For each recipe, at least 20 cylindrical samples (ϕ 27 mm × height 55 mm) were cast to perform physical and mechanical tests, 3 cylindrical samples (ϕ 27 mm × height 40 mm) were prepared to determine the bulk density and water absorption, and 3 samples 25 × 25 × 285 mm with a gauge length of 250 mm were prepared to evaluate the dimensional changes in the hardened pastes.

**Figure 4:**
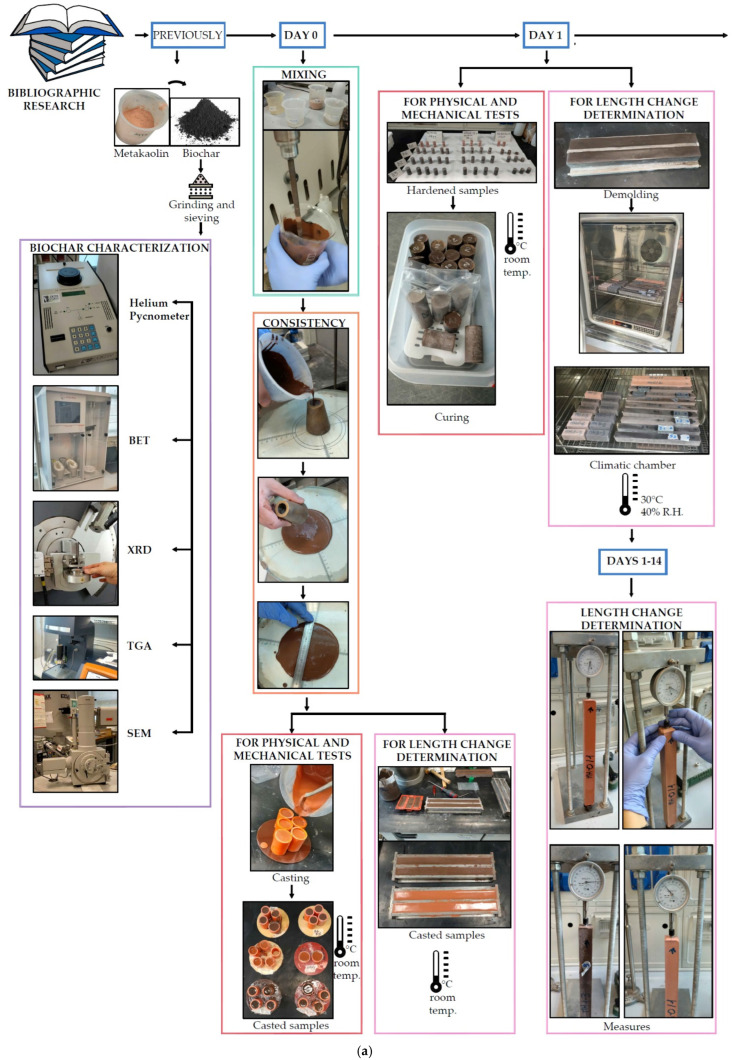

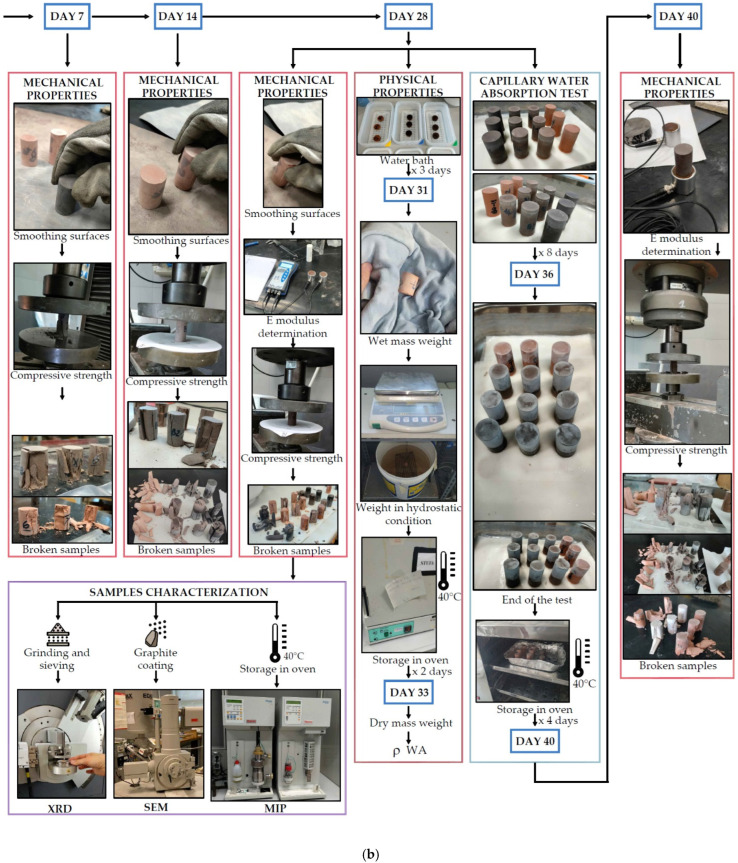
(**a**,**b**) Flowchart of the biochar characterization and AAM paste preparation and characterization.

Samples were then stored at room temperature and sealed in polyethylene bags. After 24 h, the cylinders were demoulded and stored in hermetic polypropylene containers until testing. Figure 4 shows a flowchart of the materials’ characterization, sample preparation, curing, and investigated tests at both fresh and hardened states.

### 2.3. Methods

#### 2.3.1. Determination of Consistency

Fresh pastes immediately after the preparation were cast in a brass truncated conical mould (ϕ 20–40 mm, 60 mm height). After the removal of the mould, two perpendicular diameters of the collapsed paste were measured. Standard consistency (C) was calculated as follows according to Equation (1):

C = 100·(d_m_ − d_0_)/d_0_, (1)

where d_m_ is the average diameter of the two perpendicular diameters and d_0_ is the lower diameter of the mould (i.e., 40 mm).

#### 2.3.2. Physical Properties

The bulk density (ρ_b_) of the pastes was determined by weighing the cylinders in dry, wet, and hydrostatic conditions, as prescribed by the EN 772-13 Standard [42] after 28 days of curing at environmental temperature. In particular, samples were initially submerged in water for about 3 days and the weights in wet and hydrostatic conditions were measured. Samples were later moved in an oven at 40 °C for at least 2 days before measuring the dry mass. For the three weights, different measures were performed until weight changes of less than 2% were achieved. The values of weight measured for the determination of the bulk density were also used to calculate the water absorption (WA) at atmospheric pressure according to the EN 772-21 Standard [43] at 28 days of curing.

A capillary water absorption test (EN 15801 [44]) was performed using three cylindrical samples (ϕ 27 × height 55 mm) after 28 days of curing. The amount of water absorbed was calculated using Formula (2):

Q_i_ = (m_i_ − m_0_)/A, (2)

where Q_i_ is the amount of absorbed water at time t_i_ (kg/m^2^), m_i_ is the weight of the sample at time t_i_ (kg), m_0_ is the weight of the dried sample (kg), and A is the absorption area in contact with water (m^2^).

#### 2.3.3. Pore Size Distribution

After the compression test, performed after 28 days of curing, some fragments of the broken samples were collected. The external surfaces were removed using a chisel and samples near 1.0 g were obtained. Later, the samples were dried in an oven at 40 °C for 24 h. The pore size distribution of the fragments was obtained using a mercury intrusion porosimeter (MIP Pascal 140 Series and Pascal 240 Series, Thermo Scientific, Waltham, MA, USA).

#### 2.3.4. Mechanical Properties

Compressive strength (σ_c_) was measured on cylinders (ϕ 27 × height 55 mm) initially after 7, 14, and 28 days of curing at room temperature and relative humidity of 60 ± 10%. After 28 days of curing, three cylinders were placed for 8 days in a container with the base covered with filter paper and a thin layer of water at the bottom to perform capillary testing as specified in the previous point (Section 2.3.2) but also to create an environment suitable for the development of efflorescence. After 4 days in the oven at 40 °C (40 days of total curing time, 28+8+4 days), the compressive (σ_c_) strength was measured again to compare the decrease in strength due to the efflorescence formation. The apparatus used to perform the tests was 100 kN Wolpert equipment (Wolpert, Neu Ulm, Germany) with a 5 mm/min displacement rate. Except for the dimensions of the samples, the tests were performed according to the EN 196-1 Standard [45]. Before measuring the compressive strength after 28 and 40 days, the dynamic elastic modulus (E_d_) was measured using an ultrasonic pulse velocity tester (Matest C369N, Matest, Treviolo, Italy) according to EN 12504-4 [46].

#### 2.3.5. Microstructure

After the compression test, some samples were collected and investigated with an SEM XL20 type (FEI Instruments, FEI, Hillsboro, OR, USA), a scanning electron microscope equipped with an energy-dispersive spectroscopy EDS X-ray detector. To ensure surface electrical conductivity, the fractured surfaces of the samples were coated with graphite. During the observations, an accelerating voltage of 20 kV was applied.

#### 2.3.6. Determination of Length Change

To evaluate the dimensional changes in the hardened pastes, three samples 25 × 25 × 285 mm with a gauge length of 250 mm were prepared for each recipe. After 24 h stored at environmental temperatures in plastic bags, the prisms were demoulded and placed in a climatic chamber (DY430, Angelantoni, Massa Martana, Italy) at 30 °C and 40% RH until the time of the measurement. The condition was selected to simulate demanding environments. The change in length at *x* age (L) was calculated using the following formula (4), according to ASTM C 490/C 490M–08 [47]:

L = (L_x_ − L_i_)·100/G, (3)

where L is the change in length at *x* age (%), L_x_ is the comparator reading of specimen at *x* age minus the comparator reading of reference bar at *x* age (mm), L_i_ is the initial comparator reading of specimen minus initial comparator reading of reference bar (mm), and G is the nominal gauge length (250 mm).

## 3. Results and Discussion

In Table 3, the average diameter measured during the standard consistency test (d_m_), the final consistency (C), and the physical properties (ρ_b_ and WA) are reported.

As can be observed, the consistency (C) of the samples decreases as the percentage of biochar increases owing to the fact that biochar, according to its irregular shape and porosity, absorbs water from the pastes, increasing the mixture viscosity, although even at the highest content, the paste could be cast without the creation of high-range porosities. Concerning water absorption (WA), the results are substantially similar for small amounts of biochar, but for higher percentages (5 wt% and 8 wt%), an increase in water absorption is observed due to the porosity of the biochar. For the same reason, a decrease in the bulk density is observed (near 3.5%).

**Figure 5:**
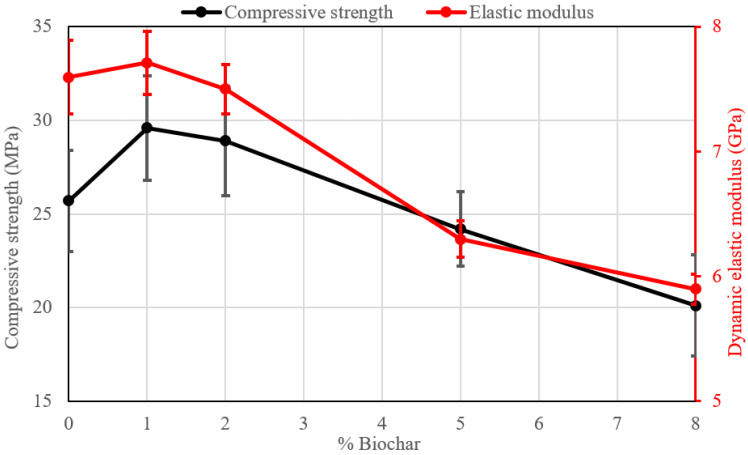
Compressive strength and dynamic elastic modulus after 28 days.

Table 4 shows the values of the mechanical properties (σ_c_) of the pastes after 7, 14, and 28 days. The development of strength takes place mainly after the first curing time while afterwards, the strength increase is low or even absent, confirming the different behaviour of AAMs when compared to traditional Portland composites. Figure 5 shows the graphics of the compressive strength of the samples after 28 days. As can be observed, the maximum values of strength at different curing times are obtained for samples with the addition of 1 wt%, which is similar to what has been found in mortars obtained with ordinary Portland cement with the addition of biochar [13]. In particular, for sample MH-1, the increase in strength after 28 days is near 15.1% compared to the reference sample. By increasing the amount of biochar (>2%), a reduction in the mechanical properties, is however observed.

Concerning the dynamic elastic modulus after 28 days (Table 4 and Figure 5), the MH-1 series with the addition of 1% of biochar shows an increase in this parameter (+0.1 GPa with respect to the reference sample). The two different mechanical properties show the same trend as a function of the biochar content (Figure 5). The increase in the modulus at 1% substitution is noteworthy since the addition of an organic, porous phase should indeed lead to lower values as the test progressively takes place at the higher content. Indeed, it has also been proposed that organic substances can participate in the development of the alkali-activated structure as will be discussed afterwards.

**Figure 6:**
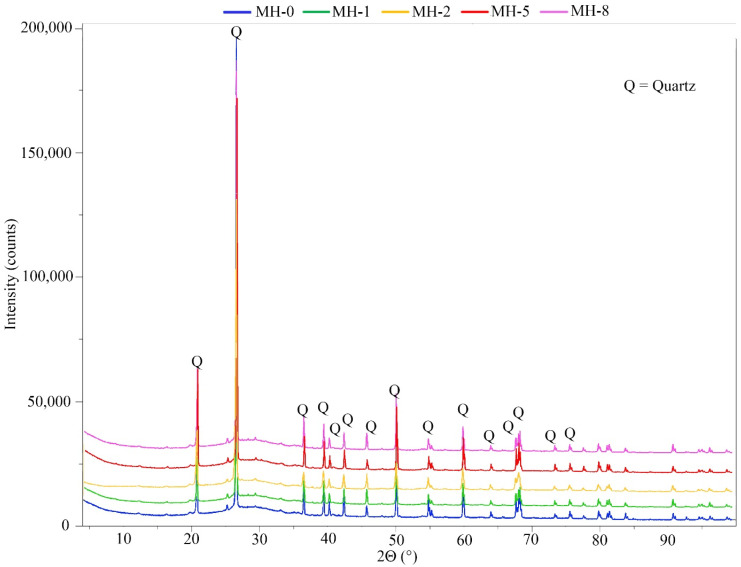
XRD graphic of the hardened pastes.

The XRD graphic of the hardened pastes is shown in Figure 6. As it can be observed, only quartz (deriving from the original metakaolin, as commented previously) is detected for all the samples and all the diffractograms have a similar shape. The calcium carbonate present in the biochar could not be detected owing to its small amount, inferior to the detection limit of the instrument (near 2%).

**Figure 7:**
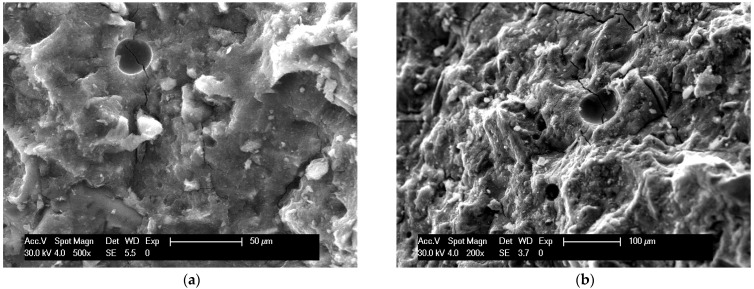

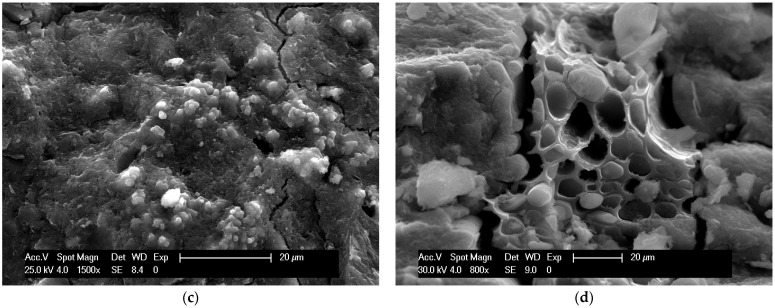
SEM images of samples: (**a**) dense and compact microstructure of sample MH-0; (**b**) morphology of MH-2; (**c**) sodium silicate crystals on surface of MH-5 sample; (**d**) biochar particle on MH-5 disclosing porosity and imperfect adhesion.

The images obtained with scanning electron microscopy on the fractured surfaces of samples cured for 28 days show a dense and compact microstructure for MH-0 samples (Figure 7a) that assures high values of compressive strength. No detectable differences can be observed at 1 and 2% biochar addition (Figure 7b), while at higher concentrations, small crystals of sodium silicate were also detected on the matrix, homogeneously arranged in the sample (Figure 7c). Some cracks can be observed (Figure 7d) close to the largest biochar particles owing to the imperfect adhesion to the matrix (Figure 7d).

**Figure 8:**
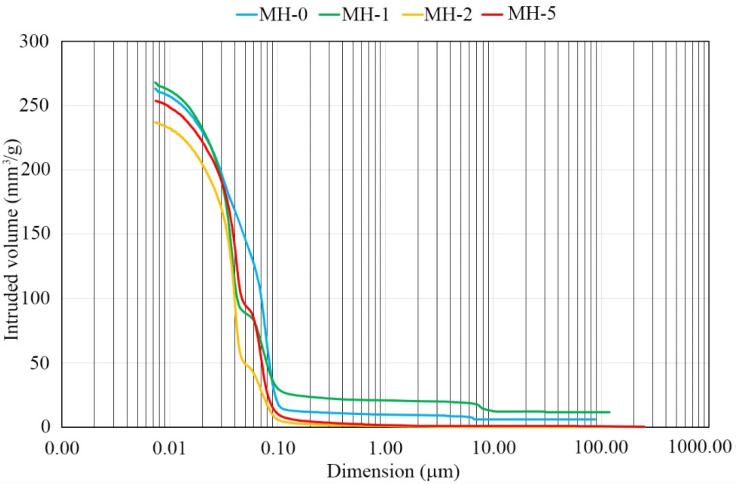
MIP results.

The results of the MIP tests are shown in Figure 8. The MH-0 unmodified sample shows a steady increase in mercury intrusion leading to high values of overall intrusion. For the modified samples, a hump in the curve is present in the porosity range from 0.04 to 0.07 μm. At 1% biochar amount, the high-range porosity (>10 μm) is not reduced compared to the MH-0 sample but is lower at the higher concentrations (2 and 5%). The detected hump can be related to the presence of biochar.

**Figure 9:**
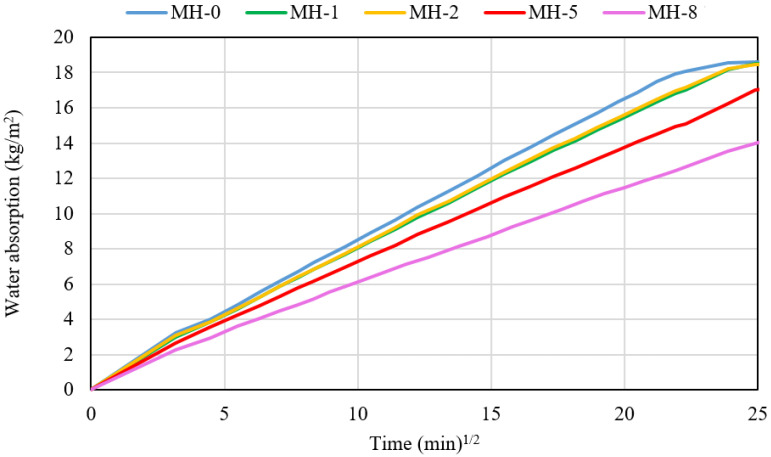
Water absorption by capillary curves.

The results of the capillary water absorption are summarized in Figure 9 where the amount of absorbed water is plotted as a function of the square root of time. Mostly because of the hydrophobic character of biochar, a progressive decrease in the rate of water permeation is observed and it is stronger as the biochar amount becomes higher than 2 wt%. This effect suggests an increased resistance to the diffusion of ions inside the modified materials, an effect that can lead to increased durability as proposed for other modified geopolymers showing high hydrophobic behaviour [48].

**Figure 10:**
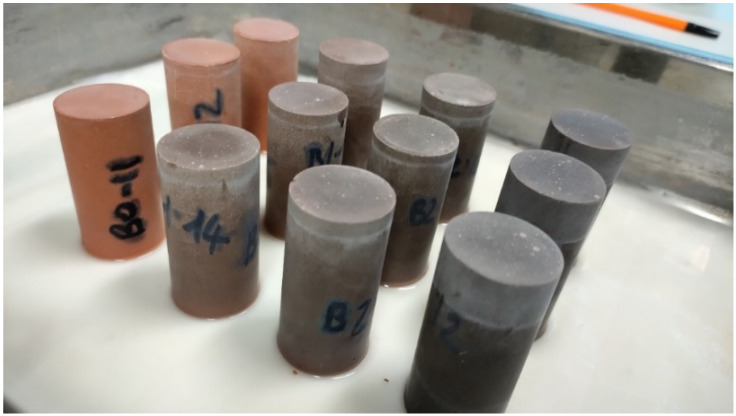
Samples during the capillary test (according to EN 15801).

During the capillary test (Figure 10), samples presented a very fine whitish patina all around the line of the capillary rise in the water (white collar). This patina generally disappeared after 8–10 h. This condition was also detected and analyzed by Manzi [49] and Zhao [31]. The first author, using the X-ray diffraction technique, found that the main element of the efflorescence was sodium carbonate hydrate; Zhao, using SEM and energy-dispersive X-ray spectroscopy (EDS), found sodium carbonate. The amount of efflorescence decreases as the biochar content increases. A similar effect was found by Zhao [31] although in systems that did not contain biochar.

**Figure 11:**
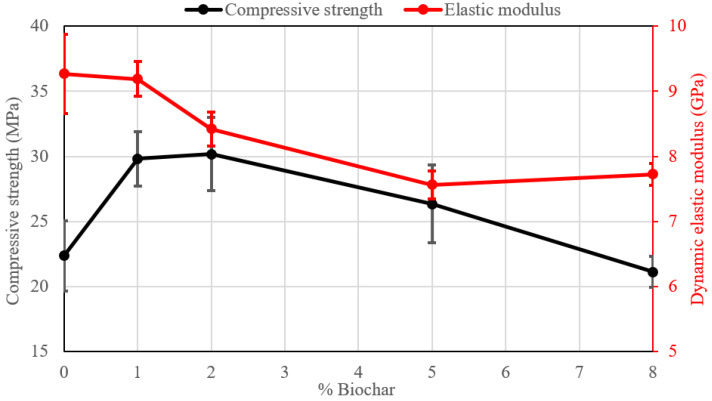
Strength and dynamic elastic modulus after the capillary test.

In order to study the effect of efflorescence formation on the materials’ properties, the samples were submitted to eight days of curing in the same condition as the capillary test. Afterwards, they were dried in an oven at 40 °C until constant weight was obtained. At this point, they underwent a mechanical test. Table 5 and Figure 11 show their compressive strength and the dynamic elastic modulus. The same trend previously observed at 14 and 28 days is obtained even at this time. The maximum values of compressive strength are detected for 1 wt% and 2 wt% of biochar, respectively, as 29.8 MPa and 30.2 MPa. In particular, comparing the values of strength in the conditions previously explained to the strength after 28 days (Δσ), it can be observed that the addition of biochar always increased the compressive strength with respect to the reference sample MH-0 that, on the contrary, decreased its strength by approximately 13%. The increase may be due to the 40 °C treatment but it is still remarkable that only the modified samples show no damage effects as it is conversely observed in the plain composition.

**Figure 12:**
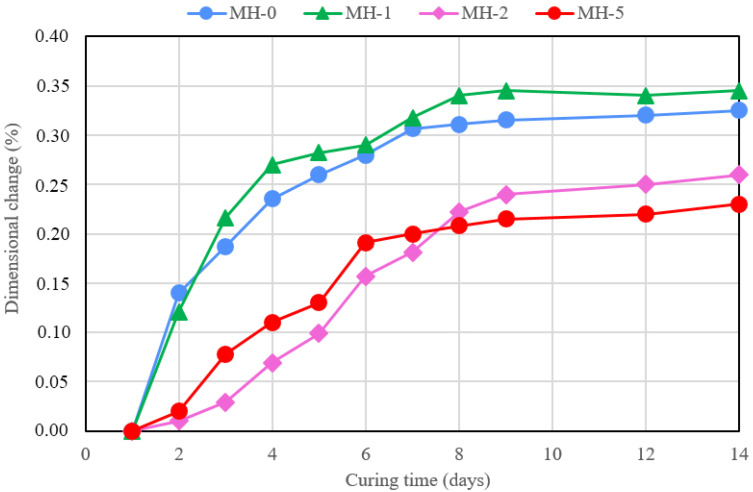
Dimensional change (%) for samples cured at 30 °C and 40% R.H. vs. curing time after the mixing stage.

Eventually, Figure 12 shows the results of the dimensional stability test performed at 30 °C and 40% R.H. A complex behaviour is observed. The lowest amount of biochar does not provide any increase in the stability of the paste, while at higher concentrations, the filler, behaving as a humidity reservoir, manages to reduce shrinkage. The effect is not, however, proportional to the filler amount.

The addition of small amounts of biochar changes the properties of the materials in a complex way. The porosity of the filler tends to decrease workability and affects the mechanical properties. On the contrary, it seems to behave as a buffer, able to control the alkalinity inside the material, reducing the negative effects of the efflorescence and mitigating, at proper contents, the dimensional changes. Indeed, biochar has an intrinsic alkaline character as proved in [33] and its porosities can behave as an alkaline reservoir that promotes and maintains the integrity of the 3D structure. The chemical character of the filler plays, however, a different role. Some studies [50] have underlined how carbon-based materials can enhance the reactivity of the binders, a feature that can explain the increase in mechanical properties at low content. This behaviour is obtained to handle the reduction in metakaolin, which is much more reactive towards alkalis than biochar and contains only a small fraction of the reactive inorganic phase. When the organic matter increases, metakaolin depletion and possibly a mismatch in the oxide ratios of the mix prevail. Moreover, the larger particles of biochar disclose an imperfect adhesion to the surrounding matrix and can create weak points in the structure, lowering the mechanical properties. The different water affinities of biochar enable the modified samples to absorb water at lower rates and to be less affected by the damages produced during its absorption, mainly efflorescence formation and leaching. After the water diffusion and efflorescence formation, while a decrease in the strength of the MH-0 series with unmodified metakaolin is detected, in the modified materials, where more constant alkalinity is possibly provided as previously underlined, the mechanical properties are maintained.

## 4. Future Scope

A more complete set of tests and experimental techniques is, however, necessary to support this initial hypothesis, i.e., the determination of the effective pH values in the bulk of the material throughout the curing processes, the analysis of the chemical structure of the gel by infrared, energy-dispersive X-ray analysis and solid-state NMR. This, however, exceeds the scope of the present paper which only focuses on the macroscopic behaviour of the modified material. Moreover, another important point is to evaluate the effects of the different chemical compositions of biochar coming from other agricultural waste.

## 5. Conclusions

In the present research, starting from an in-depth analysis of the existing literature dealing with the use of biochar in traditional (OPC) and innovative sustainable (AAMs) building materials, the addition of small amounts of biochar to metakaolin alkali-activated pastes is studied. The research focuses on the effects of biochar on macroscopic properties (physical, mechanical, microstructural, and durability) of the alkali-activated materials. The following results of the experiments can thus be summarized:

- An improvement in the mechanical properties (compressive strength and elastic modulus) is obtained with the addition of 1% of biochar (+15% for compressive strength and +0.9% for dynamic elastic modulus after 28 days). A higher amount progressively tends to decrease the mechanical performance.
- Water permeability tends to decrease with the addition of biochar, while the formation of efflorescence is reduced. An increased durability of the modified materials can thus be forecast.
- At compositions above 2%, biochar provides higher dimensional stability to the samples, a feature that was never tested before.

A tailored addition of biochar to the alkali-activated composite material can thus increase a selected property while further decreasing the environmental impact of the construction material. Future work will concentrate on the detailed effect of biochar on the chemistry of the alkali-activated 3D structure.

## Figures and Tables

**Table 1:** Summary of the literature concerning the use of biochar on building materials.

		Mixture			Binder				Biochar				Test Performed											
Author	Ref.	P	M	C	OPC	MTK	FA	GGBS	Sludge	Rice husk-Bag.	Wood -Nut	OAB (%)	Flow	σ_c_-σ_f_	σ_c_ Max MPa	SEM EDX	XRD	FTIR	TGA DSC	Perm. Absor.	*ρ*	MIP BET	LC	Dur.
Chen	[4]											5			37									
Barbhuiya	[8]											1-5			35									
Malijaee	[11]											2			70									
Chen	[12]											2			65									
Gupta	[13]											1-2			65									
Gupta	[14]											1-2			70									
Gupta	[15]											1-2			65									
Wang	[16]											1-2			70									
Muthukrishnan	[17]											20			70									
De Carvalho	[18]											1-2			73									
Zeidabadi	[19]											5			55									
Navaratnam	[20]											5			38									
Marathe	[21]											2-10												
Piccolo	[28]																							
Egodagamage	[30]											2			43									
Zhao	[31]											50			72									
Prabahar	[32]											1.5			22									
Rajamma	[34]											60			38									
Abdulkareem	[35]											10			60									
Ishak	[36]											5			41									
Yadav	[37]											55			25									
Rajan	[38]											33			65									
Saloni	[39]											90			60									
Singh	[40]											10			30									
Silvestro	[2]											10			83									
Ref. = Bibliographical Reference P = Paste M = Mortar C = Concrete OPC = Ordinary Portland cement MTK = Metakaolin FA = Fly ash GGBS = Ground granulated blast-furnace slag						Bag. = Bagasse Nut = Nuts and coconut OAB = Optimum Amount of Biochar σ_c_ = Compressive strength σ_f_ = Flexural strength σ_c_ Max = Maximum value of compressive strength (MPa) SEM-EDX = Scanning electron microscope energy dispersive X-ray Spectroscopy XRD = X-ray diffraction FTIR = Fourier transform infrared spectroscopy									TGA = Thermogravimetry DSC = Differential Scanning Calorimetry Perm. = Permeability Absor. = Absorption *ρ* = Density MIP = Mercury Intrusion Porosimetry BET = Brauner -Emmet-Teller instrument LC = Length change determination Dur. = Durability tests 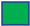 = perfomed									

**Table 2:** The recipes of the AAM pastes tested.

Name	Metakaolin (%)	Biochar (%)	Na_2_SiO_3_ (%)	NaOH (%)	H_2_O (%)
MH-0	56.02	0.00	21.29	21.29	1.40
MH-1	55.46	0.56	21.29	21.29	1.40
MH-2	54.90	1.12	21.29	21.29	1.40
MH-5	53.22	2.80	21.29	21.29	1.40
MH-8	51.54	4.48	21.29	21.29	1.40

**Table 3:** Consistency and physical properties of biochar–AAM pastes.

Sample Name	d_m_ (mm)	C (%)	ρ_b_ (Mg/m^3^)	WA (%)
MH-0	120	200	1.42 ± 0.01	27.5 ± 0.1
MH-1	117	193	1.42 ± 0.02	27.5 ± 0.7
MH-2	115	188	1.42 ± 0.01	27.5 ± 0.4
MH-5	102	155	1.41 ± 0.02	28.1 ± 0.7
MH-8	96	140	1.37 ± 0.01	28.6 ± 0.3

d_m_ = average diameter measured during the standard consistency test; C = consistency; ρ_b_ = bulk density; WA = water absorption.

**Table 4:** Mechanical properties of biochar–AAM pastes after 7, 14, and 28 days.

Sample Name	σ_c_ 7 Days (MPa)	σ_c_ 14 Days (MPa)	σ_c_ 28 Days (MPa)	E_d_ 28 Days (GPa)
MH-0	23.6 ± 3.0	26.6 ± 2.9	25.7 ± 2.7	7.6 ± 0.3
MH-1	28.3 ± 3.0	30.0 ± 2.4	29.6 ± 2.8	7.7 ± 0.3
MH-2	27.6 ± 2.8	30.4 ± 2.8	28.9 ± 2.9	7.5 ± 0.2
MH-5	21.1 ± 2.8	23.6 ± 2.2	24.2 ± 2.0	6.3 ± 0.1
MH-8	18.2 ± 2.6	19.2 ± 2.5	20.1 ± 2.7	5.9 ± 0.1

σ_c_ = compressive strength; E_d_ = dynamic elastic modulus.

**Table 5:** Mechanical properties of biochar–geopolymer pastes after 40 days, where Δσ is the decrease (−) or increase (+) of strength with respect to the 28 days of curing.

Sample Name	σ_c_ (MPa)	E_d_ (GPa)	Δ (%)
MH-0	22.4 ± 2.7	9.3 ± 0.6	−12.8
MH-1	29.8 ± 2.1	9.2 ± 0.3	+0.7
MH-2	30.2 ± 2.8	8.4 ± 0.3	+4.5
MH-5	26.3 ± 3.0	7.6 ± 0.2	+8.7
MH-8	21.1 ± 1.2	7.7 ± 0.2	+5.0

## Data Availability

The authors declare the availability of the data reported in this paper.
